# Prevalence and associated factors of complementary and integrative medicine use in patients afflicted with COVID-19

**DOI:** 10.1186/s12906-022-03722-x

**Published:** 2022-09-30

**Authors:** Mohammad Mahdi Parvizi, Sedigheh Forouhari, Reza Shahriarirad, Sepehr Shahriarirad, Ryan D Bradley, Leila Roosta

**Affiliations:** 1 Molecular Dermatology Research Center, Shiraz University of Medical Sciences, Shiraz, Iran; 2 Research Center for Traditional Medicine and History of Medicine, Shiraz University of Medical Sciences, Shiraz, Iran; 3 Infertility Research Center, Shiraz University of Medical Sciences, Shiraz, Iran; 4 Thoracic and Vascular Surgery Research Center, Shiraz University of Medical Sciences, Shiraz, Iran; 5 Student Research Committee, Shiraz University of Medical Sciences, Shiraz, Iran; 6 Helfgott Research Institute, National University of Natural Medicine, Portland, OR USA; 7 Herbert Wertheim School of Public Health and Human Longevity Sciences, University of California, La Jolla, San Diego, CA USA; 8 Australian Research Centre in Complementary and Integrative Medicine, University of Technology Sydney, Ultimo, NSW Australia; 9 Health System Research, Vice-Chancellor of Health, Shiraz University of Medical Sciences, Shiraz, Iran

**Keywords:** COVID-19, Complementary and alternative medicine, Herbal remedies, Traditional medicine, Alternative medicine, Prevalence

## Abstract

**Background:**

Complementary and Integrative Medicine (CIM) is often taken up by individuals seeking relief from different diseases. This study investigates the prevalence and associated factors of CIM use in patients with COVID-19.

**Methods:**

In this telephone-based, cross-sectional study, data on CIM usage were collected from COVID-19 patients from February till June 2020 in Fars province, Iran using a researcher-made checklist. Additionally, we asked about the patients’ attitudes toward these treatments.

**Results:**

Out of 453 patients diagnosed with COVID-19, 400 (88.30%) responded to our calls and agreed to participate in the study. Among them, 276 patients reported using CIM to treat COVID-19 [prevalence: 69% (95% CI: 64.2 to 73.5)]. The most frequently used herbal medicine among COVID-19 patients was ginger (n = 273, 98.9%), thyme (n = 263, 95.3%), and black cumin (n = 205, 74.3%). Most of these patients were recommended to use herbal medicine by their families and friends (n = 96, 34.8%). Univariable logistic regression revealed that age under 50 years old, residency in urban areas (including the capital of the province and small cities), employment, academic education, and being an outpatient were statistically significant factors resulting in CIM usage. Multivariable logistic regression revealed that CIM use among outpatients was 3.65 times more than among inpatients. In addition, patients under 50 years old used CIM 85% more than older patients. Ultimately, only 9 (3.3%) patients consulted with their doctors regarding these medications. No side effects due to CIM use were reported.

**Conclusion:**

Many patients with COVID-19 used CIM, but few consulted with their physicians in this regard. Therefore, physicians should ask their patients about CIM usage, and patients should also report their use of CIM therapies during their medical visits. Furthermore, age and hospitalization status affected CIM use among patients with COVID-19.

## Background

A new coronavirus was identified at the end of 2019, known as the severe acute respiratory syndrome coronavirus 2 (SARS-COV-2), responsible for coronavirus disease 2019 (COVID-19). This virus has infected nearly 240 million people globally, and over 5 million cases of death have been reported [1]. While the efforts to find effective treatments for COVID-19 continue, some people have opted to use complementary and alternative medicine (CIM) to prevent or treat COVID-19. Although various definitions of CIM exist, in this article, practices or products falling out of conventional medicine are considered as CIM [2].

In general, CIM is considered safe, especially in India, China, and Iran [3, 4]. Traditional Persian medicine (TPM) is one of CIM’s oldest and most popular branches. In this regard, TPM is a holistic branch of medicine that has roots in humoral medicine and has been practiced for around 7,000 years. The Islamic Golden Age was a glorious era in history when many learned people, such as Avicenna (980–1037 AD), made considerable contributions to the advancement of medicine [5–7]. According to sages of TPM, innate heat is described as a necessary component for all of the body’s organs to complete their tasks. Therefore, innate heat’s weakness could render individuals susceptible to acute and chronic diseases [8]. On the other hand, lung inflammation, cough, and dyspnea are common manifestations of COVID-19 [9]. These signs and symptoms are described as “*Zigh Alnafas*” (shortness of breath), “*Sorfeh*” (cough), and “*Tab and Homma* ” (fever) in TPM sources [10]. In this regard, the sages of TPM believed the accumulation of some thick and viscous materials in the lungs to be the main cause of cough and dyspnea. Therefore, medicine or therapy that increases innate heat while also reducing lung inflammation and swelling will prevent the disease’s progression [8, 11].

Plants have been commonly used for medicinal purposes for over 5,000 years [12, 13]. Many studies have evaluated the effect of herbal medicine and other complementary therapies for COVID-19 [14–16]. Plants such as butterbur (*Petasites hybridus*), German chamomile (*Matricaria recutita*), *Echinacea* spp., and many others have been supported by scientific studies to alleviate respiratory disease symptoms [14, 17, 18]. Furthermore, some traditional Chinese herbal medicine has been seen to possess antiviral effects, possibly inhibiting viral particles’ proliferation [19–21]. Such medication was reported to have successfully treated severe acute respiratory syndrome (SARS) during its epidemic in 2002 [15, 22, 23]. Another study reported immunomodulatory effects of some herbs such as Henna (*Lawsonia alba*), eastern purple coneflower (*Echinacea purpurea*), Ceylon leadwort (*Plumbagozeylanica* ), and velvetleaf (*Cissampelos Pareira*) in enhancing the immune system and, possibly, protecting the body against SARS-CoV-2 [15].

To the best of our knowledge, the prevalence and associated factors of CIM use in patients with COVID-19 have not been studied in Fars province, Iran. This study aims to evaluate the prevalence and associated factors of CIM use (particularly herbal remedies) by patients with COVID-19 in Fars province, Iran.

## Materials and methods

### Study design, participants, and sample size

The study population of this telephone-based cross-sectional study consisted of all patients with COVID-19 confirmed by polymerase chain reaction (PCR)-based testing who were registered in the central electronic laboratory panel for registration of COVID-19 patients in the Fars province from the 20th of February till the 20th of June 2020. This registry belongs to the Vice-Chancellery for Health of Shiraz University of Medical Sciences, Shiraz, Iran.

WinPepi® software (Copyright J.H. Abramson, Aug. 23, 2016, Version 11.65) was used for minimum sample size calculation. Having run the software, the item “DESCRIBE (descriptive epidemiology)” was selected. In the next step, the item of “K: Sample size (to estimate proportion/rate/mean, or find cases)” was selected. Finally, the item “Estimating a proportion” was selected, the considered values were recorded in front of the appropriate boxes, and the “Run” bottom was pressed. In this regard, according to a previous study [23], the minimum sample size was estimated as 350 patients with a consideration of p = 0.761, an acceptable difference of 0.05, and an expected loss of subjects of 20%.

### Inclusion and exclusion criteria

Participation criteria for the survey included symptomatic infection with SARS-CoV-2 proven by positive PCR results without any age restriction. On the other hand, patients who did not answer our phone call three times and those who rejected our invitation to participate in the study were excluded.

### Survey instrument

Because there was no suitable instrument to measure the prevalence of CIM use among patients with COVID-19, we formulated a researcher-made checklist for this study. This checklist was prepared based on consultations with experts along with a literature review using electrical databases [3, 24] and sources of TPM including *Teb-e-Akbari, Eksir-e-Azam*, and *The Canon of Medicine*, etc. The checklist was made in the Persian language in three parts. The first part included demographic characteristics such as age, sex, marital status, and duration of illness; the second part asked about the CIM that the patients used as treatment for COVID-19; and the third part asked about their experience with CIM, side effects during or after their treatment, as well their attitude toward CIM.

The final version of the instrument included 20 items. To confirm the instrument’s appropriateness, relevance, and clarity, the face validity and content validity were checked and approved by five experts in the fields of TPM and epidemiology. Furthermore, a pilot test was conducted on a sample of 30 patients to examine the instrument’s reliability. In this regard, the internal consistency was confirmed with a Cronbach’s alpha coefficient of 0.933 in the pilot study.

### Data collection

A list of patients with positive polymerase chain reaction (PCR) tests for COVID-19 in the Fars province was obtained from the specified registry. We used simple random sampling to select the patients to enroll in the study. Initially, 453 patients with COVID-19 were enrolled. For quality assurance of the data collected, a thoroughly trained interviewer was employed to call the participants, ask them the questions, and then fulfill the checklist. The questions for pediatric patients were asked of the patient’s parents or guardians. Overall, 400 (88.3%) individuals who had recently experienced COVID-19 responded to our questions and were included in the data analysis.

### Outcome measurement

Estimating the prevalence of CIM use among patients with COVID-19 was the main measured outcome in this study. The underlying factors that might affect this prevalence (according to a literature review and expert opinion) were also evaluated and analyzed using logistic regression models.

### Ethics approval and consent to participate

The protocol of this study was approved by the Ethics committee of Shiraz University of Medical Science (Ethical code: IR.SUMS.REC.1399.085, link: https://ethics.research.ac.ir/EthicsProposalView.php?id=127049 ). Enrolled patients were called and informed about the purpose of the study, and verbal informed consent was taken from the patients to participate in the study. Because this study was a telephone-based survey, taking the written informed consent was not applicable. Patients who did not consent to complete the questionnaire were not enrolled. In addition, the patients’ information was recorded anonymously. Moreover, the study complied with the guidelines of the Helsinki Declaration.

### Data analysis

IBM SPSS software version 22 (IBM SPSS Inc., Chicago, IL, USA) and Stata software version 14.2 (StataCorp LLC, College Station, Texas, USA) were used for statistical analysis. The data distribution was assessed using the Kolmogorov-Smirnov test. After confirmation of the parameters’ normal distribution, data analysis was conducted using an independent sample student’s t-test. Descriptive statistics were reported as frequency, percentage, mean data distribution, and standard deviation (SD). Moreover, univariable logistic regression was used to estimate the effect of probable underlying factors (according to the literature and expert opinion) on the prevalence of CIM use among patients with COVID-19. Then, multivariable logistic regression was applied to adjust the effect of variables with a P-value less than 0.2 in univariable logistic regression. The odds ratio (OR) and its 95% confidence interval [25] was reported. A P-value equal to or less than 0.05 was considered statistically significant at a 95% confidence interval.

## Results

### Demographic characteristics and their influence on CIM use

Out of 453 patients who had been afflicted with COVID-19 and were contacted, a total of 400 patients completed this study. The response rate of the patients was 88.3%. Among the participants, 211 (52.75%) patients were men, and 189 (47.25%) were women. On the other hand, 241 (60.25%) were outpatients, while 159 (39.75%) experienced hospitalization during their disease. Among the patients, 276 (69%) reported using CIM to treat COVID-19 (95% CI: 64.2 to 73.5). Table 1 shows the demographic characteristics of the participants based on CIM usage. Patients who used CIM to treat COVID-19 were significantly younger (46.79 ± 12.95 vs. 57.63 ± 18.16, P < 0.001) and had academic education. In addition, the mean duration of the disease in the patients who received and did not receive CIM was 22.05 ± 6.67 days and 27.93 ± 11.27 days, respectively (P < 0.001).

**Table 1:** Demographic characteristics of individuals who experienced COVID-19, classified according to their use of traditional and integrative medicine during the disease course

Variable	Use of traditional and integrative medicine		Crude OR ^a^ (95% CI ^b^)	P-value ^c^	Adjusted OR^d^ (95% CI)	P-value
	Yes *n = 276*	No *n = 124*				
**Age**						
*≥ 50 years old*	112 (59.42)	82 (66.13)	1	< 0.001*	1	1
*< 50 years old*	164 (40.58)	42 (33.87)	2.85 (1.84 to 4.45)		**1.85 (1.07 to 3.20)**	0.029*
**Sex**; n (%)						
*Women*	129 (46.7)	60 (48.4)	1		-	-
*Men*	147 (53.3)	64 (51.6)	1.07 (0.70 to 1.63	0.496		-
**Marital status**; n (%)						
*Single*	43 (15.6)	16 (12.9)	1	-	-	-
*Married*	232 (84.1)	107 (86.3)	0.81 (0.44 to 1.50)	0.496	-	-
*Other*	1 (0.4)	1 (0.8)	-	-	-	-
**Place of residence**; n (%)						
*Rural area*	15 (5.4)	18 (14.5)	1		1	
*Capital of Province*	114 (41.3)	53 (42.7)	2.58 (1.21 to 5.51)	0.014*	0.81 (0.33 to 1.99)	0.639
*Small cities*	147 (53.3)	53 (42.7)	3.33 (1.57 to 7.07)	0.002*	1.57 (0.70 to 3.52)	0.272
**Occupation**; n (%)						
Unemployed	138 (48.91)	53 (42.74)	1	0.253	-	-
Employed	141 (51.09)	71 (57.26)	0.78 (0.51 to 1.12)		-	-
**Educational level**; n (%)						
*Under-diploma and diploma*	125 (45.29)	78 (62.90)	1		1	
*Having academic degree*	151 (45.7)	46 (37.1)	2.05 (1.33 to 3.16)	0.001*	1.24 (0.67 to 2.30	0.489
**Hospitalization**; n (%)						
*Yes*	79 (28.6)	80 (64.5)	1		1	
*No*	197 (71.4)	44 (35.5)	4.53 (2.89 to 7.19)	< 0.001*	3.65 (2.21 to 6.02)	< 0.001*

(a) OR: Odds ratio calculated using univariable logistic regression; (b) CI: confidence interval; (c) P-values equal and less than 0.05 were considered significant. (d) OR adjusted for age, place of residence; educational level, and hospitalization because of COVID-19 using multivariate logistic regression

Table 1 shows the effect of some underlying factors on the use of CIM among patients with COVID-19. Overall, 179 (71.4%) of the patients who used CIM were not hospitalized during the period of their disease (OR = 4.53, 95%CI: 2.89 to 7.19, P < 0.001). Also, the odds of CIM use in patients with academic degrees were 2.05 times higher than in those without an academic degree (OR = 2.05, 95%CI: 1.33 to 3.16, P = 0.001). Furthermore, patients under 50 years old used CIM 2.85 times more than those equal to or more than 50 years old (OR = 2.85, 95%CI: 1.84 to 4.45, P < 0.001). Moreover, patients residing in urban areas, both in the province’s capital and small cities, used significantly more CIM than those who lived in rural areas. However, multivariable logistic regression analysis revealed that being an outpatient (adjusted OR = 3.65, 95%CI: 2.21 to 0.45, P < 0.001) and the age of the patients (adjusted OR = 1.85, 95%CI: 1.07 to 3.20, P = 0.029) were the only factors that could affect CIM use among the patients with COVID-19. In this regard, the prevalence of CIM use among outpatient participants was 3.65 times more than the participants who had experienced hospitalization, while the prevalence of CIM use among the patients under 50 years old was 85% more in comparison with the older patients.

### The CIM used by patients with COVID-19

Table 2 shows the frequency of herbal remedies used by patients to treat COVID-19. As shown, the most frequently used herbal medicine was ginger (98.9%), mostly in brewed form (84.2%), followed by thyme (95.3%), also mainly in brewed form (93.2%), and black cumin (74.3%), mainly in soaked form (76.1%). Moreover, 81 (29.35%) of the participants used mixed herbal remedies, especially in powder or decoction forms.

**Table 2:** Frequency and preparation method of used herbal medicine among COVID-19 patients

Common name	Scientific name	Total	Brewed	Decoction	Soaked^£^	Powder	Essence	Inhalation	Topical
Ginger	*Zingiber officinale*	273 (98.9)	230 (84.2)	9 (3.3)	-	37 (13.6)	-	-	-
Shirazi Thymes	*Zataria multiflora* ^*¶*^	263 (95.3)	245 (93.2)	24 (9.1)	-	5 (1.9)	1 (0.3)	-	-
*Black cumin*	*Nigella sativa*	205 (74.3)	49 (23.9)	3 (1.5)	156 (76.1)	-	-	-	-
Quince seed	*Cydonia oblonga*	202 (73.2)	139 (68.8)	31 (15.3)	19 (9.4)	7 (3.5)	1 (0.5)	-	-
Garlic	*Allium sativum*	187 (67.8)	4 (2.1)	-	1 (0.05)	184 (98.4)	2 (1.1)	-	-
Jujube	*Ziziphus jujuba*	183 (66.3)	71 (38.8)	62 (33.9)	44 (24)	21 (11.5)	-	-	-
Borage	*Borago officinalis*	175 (63.4)	144 (82.3)	34 (19.4)	-	-	-	-	-
Cinnamon	*Cinnamomum verum*	157 (56.9%)	157 (100%)						
Licorice	*Glycyrrhiza glabra*	135 (48.9)	34 (25.2)	97 (71.9)	1 (0.7)	6 (4.4)	-	-	-
Violet	*Viola*	118 (42.8)	84 (71.2)	38 (32.2)	-	-	-	-	1 (0.8)
Hollyhock	*Alcea*	99 (35.9)	71 (71.7)	26 (26.3)	-	1 (0.1)	-	-	-
Chamomile	*Matricaria chamomilla*	90 (32.6)	90 (100)						
Cordyamyxa	*Cordia myxa*	61 (22.1)	18 (29.5)	43 (70.5)	2 (3.3)	3 (4.9)	-	-	-
Common wormwood	*Artemisia absinthium*	60 (21.7)	23 (38.3)	33 (55)	1 (1.7)	5 (8.3)	-	-	-
Cuscuta monogyna	*Cuscuta epithymum*	59 (21.4)	26 (44.1)	33 (55.9)	1 (1.7)	1 (1.7)	1 (1.7)	-	-
Hemp	*Cannabis*	10 (3.6)	2 (20)	4 (40)	-	-	4 (40)	-	-
Cedar	*Cedrus*	7 (2.5)	2 (28.6)	-	-	-	5 (71.5)	-	-

^¶^ Different from *Thymus vulgaris.*^£^*Soaked: Putting a herb into a bath of water for several hours to produce muslin or a high concentration of the extract*

Additionally, 13.4% of the patients with COVID-19 reported use of other kinds of CIM remedies, including wet cupping (*Hejamat*) (21 patients consisting of 10 men and 11 women), dry cupping (nine patients consisting of seven men and two women), yoga (three women), leech therapy (one man and one woman), and Islamic medicine medications (two women).

### Source of information and attitude about CIM for the treatment of COVID-19

Table 3 shows the details of information about using CIM in patients with COVID-19 including the source of information, physician oversight, and reason for withholding CIM use. Most of the patients were recommended herbal medicines by their friends and relatives (34.8%), followed by apothecaries (35.4%). According to the patients’ reports, none visited a secondary or tertiary center to receive a prescription for CIM. Only 9 (3.3%) participants had consulted with their doctors regarding CIM usage. Most participants (32.2%) did not feel the need to consult with their family physicians or conventional medical doctor. Notably, 77 (27.9%) patients believed that their doctors did not have enough knowledge regarding CIM, especially herbal remedies, to give them reasonable and useful advice. On the other hand, 54 (19.6%) of the patients reported that their family physicians or conventional medical doctors asked them about using CIM, especially herbal remedies, during the patients’ disease course. Among them, 32 (59.3%) of these physicians approved of the use of herbal medicines, while the other 22 (40.7%) did not reflect on the use of herbal medicine. Furthermore, none of the participants reported any side effects due to using herbal remedies to treat COVID-19.

**Table 3:** Evaluation of the attitude of the patients with COVID-19 about complementary and integrative medicine use for the treatment of COVID-19

Variable	Frequency; *n*	Percentage; *%*
**Method of acquaintance**		
Family and friends	96	34.8
Broadcasting news television and radio	29	10.5
Internet and social media	27	9.8
Articles, books, and magazines	10	3.6
Apothecary	95	34.4
Other	19	6.9
**Consulting with the treating physician**		
Yes	9	3.3
No	267	96.7
**Reason for not informing the physician**		
Forgetting to consult	20	7.2
Did not sense the need to inform	89	32.2
Sensed that the physician did not have enough knowledge regarding herbal and alternative medicine	77	27.9
Fear of advising against the usage of herbal medicine	68	24.6
Other	22	8
**Side effects**		
Yes	0	0
No	276	100

## Discussion

The present study found that 69% of patients who experienced COVID-19 used CIM specifically to treat this disease. Briefly put, it seems that younger people, patients with academic degrees, and patients living in urban areas used significantly more CIM to treat COVID-19. Our results align well with several studies proposing that CIM therapy has become a popular therapeutic method for preventive and treatment purposes nowadays [26–28].

Moeini et al.‘s study revealed that although 71.65% of the normal population of Babol, a Northern city in Iran, had used complementary and alternative medicine throughout their lives, only 6.21% of them visited a complementary medicine therapist to receive these medications. In addition, they demonstrated that herbal remedies were the most common complementary medicine (58.30%) among the normal population in Babol [29]. Furthermore, Montazeri et al. reported that only about one-third of the patients who suffered cancer in Tehran, Iran, used complementary and alternative medicine for their disease. In addition, their study reported that praying and spiritual health were the most common CAM used among patients with cancer [30]. On the other hand, Abolhassani et al.‘s study, which was conducted on 5,000 patients of the Iranian population who had used complementary medicine and Iranian traditional medicine, revealed that energy therapy and praying, followed by food regime and fasting were the most common types of complementary medicine that they used. Accordingly, only 11.6% of these patients preferred using herbal remedies to treat their disease [4].

Several studies have been conducted on the prevalence of CIM use in Shiraz, Fars province. For instance, Dastgheib et al. revealed that 31.3% of patients referring to dermatology clinics reported using CIM for their dermatologic conditions [31]. Moreover, Hashempur et al. found that 75.3% of the patients with diabetes mellitus in Shiraz used at least one kind of CIM during their disease period [5]. On the other hand, Molavi Vardanjani et al. demonstrated that up to 97% of lactating women used CIM in their breastfeeding period [32]. Although the prevalence of CIM use was different in these various conditions, Shiraz is the biggest province and the largest referral medical center in Southern Iran, so the results of studies conducted in Shiraz could be a reasonable representation of the population of Southern Iran areas.

In our study, certain demographic characteristics were found to play a role in the likelihood of patients using CIM, especially herbal remedies. The younger population and patients with higher educational levels were more inclined to practice this kind of medication. On the other hand, CIM usage was not associated with the sex of the patients and their marital status. A similar study suggested sex and educational level as factors associated with herbal medicine usage [33]; however, our results only agree with the latter. Moreover, most of the patients in our study who used CIM were outpatients. On the other hand, the multivariable logistic regression revealed that the most important factors affecting CIM use were being under 50 years old and being an outpatient. These findings may be explained by a lower degree of concern for drug interactions between herbal and conventional medicine among outpatients and younger patients in comparison with older and hospitalized patients. Also, it seems that outpatients usually use self-medication and traditional and home remedies, while those hospitalized are treated according to the established protocols.

Ginger (98.9%), thyme (95.3%), black cumin (74.3%), and quince seed (73%) were the most commonly used herbs among the participants in our study. The therapeutic effects of some of these herbs have been recently evaluated in the treatment of COVID-19. A study by Safa O. et al. [34] revealed that ginger could affect COVID-19 treatment by increasing the recovery rate of clinical symptoms, including cough, fever, and fatigue, as well as paraclinical features such as C-reactive protein and thrombocytopenia. In another study, luteolin, a flavonoid existing in medicinal herbs like thyme, garlic, and chamomile tea, was mentioned to contain therapeutic, in this case, antiviral, activity [35–37]. Moreover, garlic is known to have potentially healing effects against pulmonary symptoms associated with COVID-19. Also, evidence shows that certain compounds existing in garlic contain anti-inflammatory and antiviral activities [35, 38]. Ang L. et al. [39] also faced significant results regarding herbal medication’s effectiveness when integrated with conventional medicine, suggesting the likelihood of herbal remedies playing an evident role in treating COVID-19.

On the other hand, it should be considered that the effects of herbal remedies or other complementary medicines are not limited to the antivirus effect against COVID-19. Some of the herbal remedies reported by the patients in this study, such as ginger, thyme, black cumin, jujube, and licorice, have immunomodulatory, bronchodilatory, anti-inflammatory, antioxidant, antihistaminic, and antitussive activities [40–45], so they could be effective in the treatment of other viral and non-viral diseases as well.

Patients were familiarized with herbal medicine through several different methods. Over half of them were acquainted with these medications via friends, family, and apothecaries. Other sources the patients used to find herbal consumption remedies for the treatment of COVID-19 included, in order or frequency, news, television and radio, internet and social media, articles, books, and magazines. In another study, Mekuria AB et al. [46] found families and friends to be more than half of the participants’ main source for pursuing herbal remedies, followed by previous herbal medicine users, media, and traditional healers; these are in line with the results of our study. This reveals that the majority of patients using CIM earn their information from unverified sources, which may not provide enough knowledge about herbal remedies and CIM, as well as possible side effects, proper dosage for consumption, and contraindications of these medications. Although our patients did not report any side effects of herbal remedies during their COVID-19 course, evidence indicates that some side effects include diarrhea, abdominal pain, throat pain, constipation, headache, hypertension, hypotension, hypoglycemia, etc. [47]. Moreover, some documents cautioned about the long-term adverse effect of using herbal remedies, as well as drug interactions among the patients with COVID-19 [48, 49]. Therefore, it is strongly advised that patients seeking CIM consult with a reliable physician who can provide them with accurate scientific information regarding CIM, including herbal remedies.

Unsupervised consumption is a major concern regarding CIM [50]. Almost all our study participants being treated with CIM did not consult with their doctor, mostly due to either not feeling the need to mention it or not trusting the caretakers’ knowledge of traditional medicine. Similarly, other studies revealed that the majority of the participants who used CIM (often recommended by their friends and peers) did not consult with their doctor [51, 52].

It seems that raising public awareness about the possible advantages and disadvantages of herbal remedies through social media can greatly benefit people seeking this kind of treatment. Nevertheless, unsupervised use of some products containing a mixture of herbs could result in allergic reactions, toxicity, and in severe cases, organ failures [12, 53]. In this regard, making use of telemedicine, distributing text messages, preparing photos and videos to post on social media, distributing standard pamphlets and brochures, and having trained CIM practitioners present in television and radio programs are recommended to augment the knowledge, attitude, and practices of the society.

Many adverse effects have been mentioned in the literature regarding the herbal medicines used by COVID-19 patients in our study. Gastrointestinal side effects like heartburn, burping, diarrhea, and general stomach discomfort are reported in ginger users [54]. For thyme, side effects such as digestive upset, headache, and dizziness are reported [55]. Black cumin can cause allergic reactions, constipation, stomach upset, and vomiting [56]. Besides the direct side effects, using CIM may cause a delay in starting standard effective treatments for the patient. This delay can lead to poor outcomes, especially in diseases with acute or progressive courses such as COVID-19.

The most important aspect of our study was conducting it on patients in the initial period of the emergence of COVID-19. Hence, the people who used complementary medicine against COVID-19 were not affected by the results of experimental studies and clinical trials. In other words, the results of this study portray the original beliefs of the study population about the effects of traditional medicine in the prevention and treatment of COVID-19 regardless of clinical studies. Accordingly, the authors suggest designing further studies to evaluate the prevalence of CIM use among COVID-19 patients when several clinical trials have revealed the efficacy of some mono-herbal, poly-herbal, and other complementary medicine in the treatment of COVID-19.

Our study faces some limitations. First, the patients were interviewed a few weeks after their treatment; thus, the patients may be subject to recall bias. Second, the checklist used in this study was researcher-crafted, suggesting that an extended standard questionnaire might be able to provide more insight into our topic of research. Next, our study only included the first months of the COVID-19 pandemic. We recommend that the trend of CIM use for the treatment of COVID-19 be studied in a more extended time frame. Furthermore, although several forms of herbal remedies, including package form, medicine, and dietary supplements, are available in Iran, we did not distinguish between these forms. Therefore, the authors recommend evaluating the patients’ preferences to choose each form of herbal remedy in further studies. Moreover, hospitalization was not considered as an outcome of using herbal and complementary medicine in patients with COVID-19 in this study; this issue can be explored in future studies. This study focused on CIM use during COVID-19, disregarding the overall attitude and routine CIM use before the pandemic. Next, the time taken to improve was not considered an outcome of the study because the data about when the patients started their CIM use was not collected. Finally, our study was single-center in nature, while multi-centered studies may provide a broader view concerning the treatment of COVID-19 with CIM.

## Conclusions

In many cases, the use of CIM, especially herbal remedies, in COVID-19 patients is ill-advised. Ginger, thyme, black cumin, and quince seed were among the most commonly used medicinal herbs in our population of patients who experienced COVID-19. It is necessary for patients, as well as physicians in conventional medicine, to be made aware of the indications, contraindications, advantages, and side effects of CIM, especially herbal remedies, to encourage the appropriate use of these treatments.

## Data Availability

The datasets used and/or analyzed during the current study are available from the corresponding author on reasonable request.

## List of abbreviations

CIM: Complementary and integrative medicine.
COVID-19: Coronavirus disease 2019.
SARS-CoV-2: Severe acute respiratory syndrome coronavirus 2.
PCR: Polymerase chain reaction.
